# Stripe Rust Effector Pst_9302 Inhibits Wheat Immunity to Promote Susceptibility

**DOI:** 10.3390/plants13010094

**Published:** 2023-12-27

**Authors:** Haibin Zhao, Jiangyu Huang, Xiaoyan Zhao, Ligang Yu, Xiaodong Wang, Congcong Zhao, Hojjatollah Rabbani nasab, Chunlei Tang, Xiaojie Wang

**Affiliations:** 1 State Key Laboratory of Crop Stress Resistance and High-Efficiency Production, College of Plant Protection, Northwest A&F University, Xianyang 712100, China; zhaohaibinxn1934@163.com (H.Z.); hjy319329727@163.com (J.H.); zhaoxiaoyan2021@nwafu.edu.cn (X.Z.); yuligang@nwafu.edu.cn (L.Y.); xiaodongwang@nwsuaf.edu.cn (X.W.); ggbbzcc@163.com (C.Z.); 2 State Key Laboratory of Crop Stress, Plant Protection Department, Golestan Agricultural and Natural Resource Research and Education Center, Gorgan P.O. Box 49156-77555, Iran; h.rabbani@areeo.ac.ir

**Keywords:** *Puccinia striiformis* f. sp. *tritici*, effector, HIGS, VDAC1, plant immunity

## Abstract

*Puccinia striiformis* f. sp. *tritici* is an obligate biotrophic fungus that causes destructive stripe rust disease in wheat. During infection, *Pst* secretes virulence effectors via a specific infection structure—the haustorium—inside host cells to disturb host immunity and promote fungal colonization and expansion. Hence, the identification and functional analyses of *Pst* effectors are of great significance in deciphering the *Pst* pathogenicity mechanism. Here, we identified one candidate *Pst* effector Pst_9302 that could suppress Bax-triggered cell death in *Nicotiana benthamiana*. qRT-PCR analyses showed that the transcript levels of *Pst_9302* were highly increased during the early infection stages of *Pst*. The transient expression of *Pst_9302* in wheat via the type-three secretion system (T3SS) significantly inhibited the callose deposition induced by *Pseudomonas syringae* EtHAn. During wheat–*Pst* interaction, *Pst_9302* overexpression suppressed reactive oxygen species (ROS) accumulation and cell death caused by the avirulent *Pst* race CYR23. The host-induced gene silencing (HIGS) of *Pst_9302* resulted in decreased *Pst* pathogenicity with reduced infection area. The results suggest that Pst_9302 plays a virulence role in suppressing plant immunity and promoting *Pst* pathogenicity. Moreover, wheat voltage-dependent anion channel 1 protein (TaVDAC1) was identified as candidate Pst_9302-interacting proteins by yeast two-hybrid (Y2H) screening. Pull-down assays using the His-Pst_9302 and GST-TaVDAC1 protein verified their interactions. These results suggest that Pst_9302 may modulate wheat TaVDAC1 to regulate plant immunity.

## 1. Introduction

In nature, plants face threats from the environment and microbes. To defend against pathogenic microbes, plants have evolved a two-layered immune system [1]. Pattern recognition receptors (PRRs) located on plant plasma membrane perceive the conserved pathogen/damage/microbe-associated molecular patterns (PAMPs/DAMPs/MAMPs) to activate the first layer immunity, called PAMP-triggered immunity (PTI). Surface pattern recognition receptors mainly refer to receptor-like proteins or receptor-like kinases, such as FLS2 and RXEG1 [2,3]. For survival, pathogenic microorganisms secrete effectors into host cells where they overcome plant immunity, resulting in effector-triggered susceptibility (ETS) [1]. In turn, plants have further evolved resistance proteins (R), known as intracellular nucleotide-bound leucine-rich repeat receptors (NLRs), for the perception of specific avirulence (Avr) proteins via direct ligand–receptor interactions or the indirect detection of effector activity, leading to effector-triggered immunity (ETI) [4]. ETI is characterized with strong and localized hypersensitive cell death (HR) and reactive oxygen species (ROS) burst, which is particularly effective against biotrophic pathogens that are entirely dependent on living host cells. Plant immunity is a holistic system in which PTI and ETI are not independent but contribute to each other [5,6,7].

Uncovering molecular mechanisms underlying pathogen pathogenicity could lay the foundation for engineering new strategies for improving disease resistance in crop plants by preventing pathogens from regulating host plant immunity. Effectors as important pathogenicity factors have been the focus in the deciphering of pathogen pathogenicity. Effectors are expressed at different infection stages and functional in different host plant cellular components [8], which may influence various biological processes, facilitating pathogen proliferation and dispersal. Studies have shown that plant pathogen effector proteins can function to interfere with the host hormone metabolism pathway, ubiquitination pathway, gene transcription, RNA processing, HR, etc. [9,10,11,12,13,14].

*Puccinia striiformis* f. sp. *Tritici* (*Pst*), the causal agent of wheat stripe rust, devastates wheat crops worldwide [15,16]. *Pst* is an obligate biotrophic pathogen that establishes an elaborate parasitic relationship with its host plants. During infection, *Pst* forms haustoria to penetrate host cell walls, contact host cell membranes, and enable nutrient uptake [17,18]. Like many other plant pathogens, rust fungi secrete effector proteins into the host cells [19]. In spite of the importance in understanding the rust–wheat pathosystem, the lack of a stable transformation system limits the study of wheat rust fungal effectors [20]. An alternative approach for effector screening developed is using a *Pseudomonas fluorescens* (*Pf*) strain EtHAn (effector-to-host-analyzer) to deliver oomycete or fungal effectors into host cells via the bacteria type-three secretion system (T3SS) [21]. The developed host-induced gene silencing (HIGS) technique also provides a powerful tool for knocking down the expression of *Pst* effectors to study their roles in *Pst* pathogenicity [22,23]. With the sequencing of the genome and haustorium transcriptome of the stripe rust fungus, a batch of effectors have been identified and the functional mechanism of certain effectors have been characterized [24,25]. Studies show that *Pst* effectors are able to translocate into different plant cell compartments to disturb plant immunity [12,26,27,28,29]. Pst_12806 enters into wheat chloroplasts where it interacts with the cytochrome b6f complex element TaISP to affect chloroplast metabolism and ROS production, thereby promoting *Pst* infection [28]. Interestingly, another two *Pst* effectors, Pst_4 and Pst_5, also interact with TaISP in the cytoplasm to prevent TaISP from entering chloroplasts [29]. Pst_A23 enters the wheat splicesome to directly bind the splicing sites of wheat pre-mRNAs and reprogram wheat pre-mRNA alternative splicing [12]. PstGTA1 can locate in the wheat cell nucleus, which binds to the promoter region of the wheat susceptibility gene *TaSIG* to activate its transcription by regulating the acetylation level of H3K4 [30]. The unconventional effector PsSpg1 enhances the phosphorylation of the wheat susceptibility gene TaPsIPK1 to promote the nuclear entrance of TaPsIPK1, where it modulates the transcription activity of TaCBF1 and promotes wheat susceptibility [31].

In this study, from the genome of *Pst* race CYR32 [25], a candidate effector Pst_9302 that is highly induced at the early infection stage of *Pst* was identified. *Pst_9302* specifically exists in *Pst* and is conserved among different *Pst* isolates. The transient expression of *Pst_9302* inhibited *Bax*-induced cell death in *Nicotiana benthamiana*. The delivery of Pst_9302 into wheat cells via EtHAn suppressed the PTI-associated callose deposition caused by non-pathogenic bacteria. Upon avirulent *Pst* challenge, Pst_9302 overexpression inhibits wheat ETI, resulting in decreased ROS accumulation and HR occurrence. These data indicated the virulence function of Pst_9302 in suppressing host immunity, including both PTI and ETI. Furthermore, the host-induced gene silencing (HIGS) of *Pst_9302* decreased the virulence of *Pst*, resulting in reduced uredium formation and attenuated fungal infection area. The yeast two-hybrid (Y2H) assay identified that the wheat voltage-dependent anion channel 1 protein TaVDAC1 was able to interact with Pst_9302, and the interactions between them were verified via the pull-down assay. VDAC is a major outer mitochondrial membrane protein, which is reported to participate in mitochondrial-mediated apoptosis. Overall, our data suggest that *Pst_9302* is an important *Pst* pathogenicity factor which likely regulates plant immunity by targeting the mitochondrial VDAC in plants.

## 2. Results

### 2.1. Pst_9302 Is Induced during Pst Infection in Wheat

**Figure 1:**
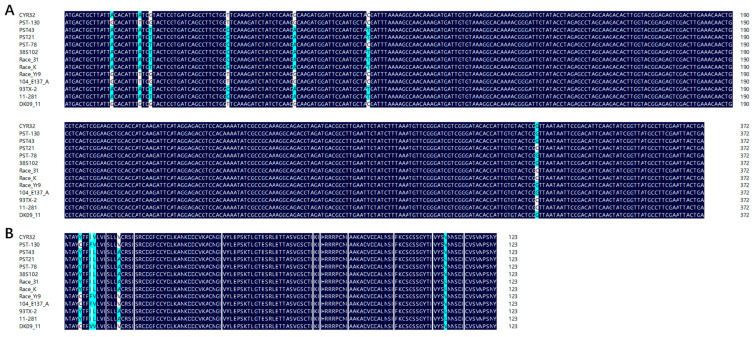
Polymorphism analyses of *Pst_9302* in 13 *Pst* isolates. (**A**). Nucleotide acid sequence alignment of *Pst_9302* in 13 *Pst* isolates. The nucleotide acid sequences were downloaded from the NCBI database. (**B**). The amino acid sequence alignment of *Pst_9302* from 13 *Pst* isolates. The blue code represents 100% similarity and cyan code represents 50% similarity. The numbers on the left represent the name of the *Pst* isolate.

**Figure 2:**
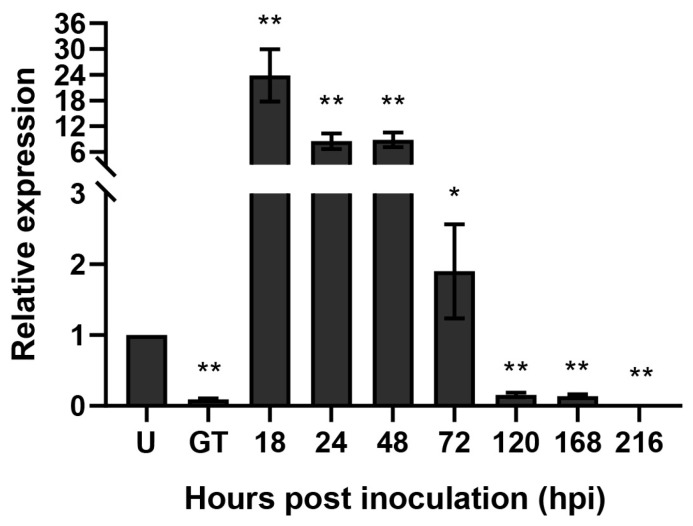
Transcript profiles of *Pst_9302* during *Pst* infection. The second leaves of wheat cultivar Swon11 were inoculated with virulent *Pst* race CYR32 and the samples were collected at 18, 24, 48, 72, 120, 168, and 216 h post inoculation (hpi). Urediniospores, U; germ tubes, GT. *Pst* elongation factor gene *PsEF1* was used as the internal control gene. Data are mean ± SD from three biological replicates. Asterisks indicates significant difference using unpaired two-tailed Student’s *t*-test via comparison to that in urediniospores (*, *p* < 0.05; **, *p* < 0.01).

From the genome of the *Pst* race CYR32, *Pst_9302* encoding a secreted protein was identified, with an open reading frame of 372 bp. SignalP 5.0 predicted that Pst_9302 contains a signal peptide encoded by the N-terminal 1–21 amino acids (Figure S1). The mature protein of Pst_9302 contains no transmembrane domain or conserved domain. BlastN analyses revealed no homologues genes in either *Puccinia triticina* or *Puccinia graminis*, the other two rust fungal pathogens in wheat, or in other pathogenic fungi. Among 13 tested *Pst* isolates from different countries, seven nucleotides in the ORF of *Pst_9302* showed variations (Figure 1A), which resulted in variations in five amino acids (Figure 1B). The results showed that *Pst_9302* is a *Pst*-specific secreted protein with low polymorphism within *Pst*. To determine whether *Pst_9302* is involved in *Pst* infection, we measured its transcript levels at different *Pst* infection stages. qRT-PCR analyses showed that *Pst_9302* was highly induced in planta infection of *Pst*, reaching the peak at 18 h post inoculation (hpi), and was 23-fold higher than that in urediniospores (Figure 2). At 18 hpi, *Pst* begins to form the initial haustorium, suggesting the role of *Pst_9302* at the early infection stage of *Pst*.

### 2.2. Pst_9302 Is a Candidate Effector That Is Able to Suppress Bax-Induced Cell Death in Planta

**Figure 3:**
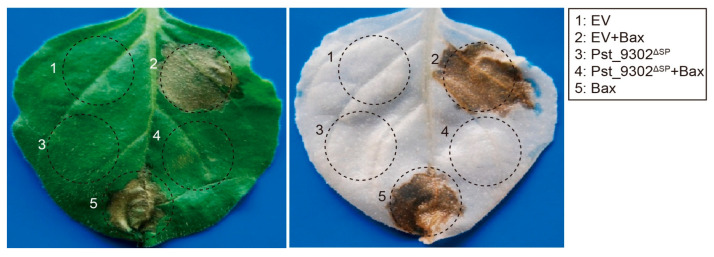
Pst_9302 suppresses Bax-triggered cell death in *N. benthamiana*. *N. benthamiana* leaves were infiltrated with *A. tumefaciens* GV3101 carrying various constructs, as labeled in the schematic drawing. For co-filtration assays, leaves were first infiltrated with *Agrobacteria* expressing Pst_9302^ΔSP^ without the signal peptide followed by infiltration with Agrobacteria expressing mouse Bax after 24 h. Photos were taken 5 days after infiltration. The empty vector (EV) pGR106 was used as negative control. The same leaf was decolorized with destained solution (right).

The mouse proapoptotic factor Bax is reported to induce programmed cell death (PCD) that is similar to HR in plants [14,32]. Thereby, detecting the suppression ability of Bax-triggered cell death has become a high-throughput and effective assay to determine the virulence function of pathogen effectors in inhibiting plant defense responses [33,34]. Here, obvious cell death was observed in *N. benthamiana* leaves expressing Bax mediated via *Agrobacterium* infiltration (Figure 3). In *N. benthamiana* leaves infiltrated with *Agrobacterium* carrying Pst_9302 without the signal peptide (Pst_9302^ΔSP^) 24 h before Bax infiltration, no cell death was observed (Figure 3). In contrast, in *N. benthamiana* leaves transiently expressing the negative control EV (empty vector), Bax-induced cell death was not affected (Figure 3). The results showed that the transient expression of Pst_9302 suppressed Bax-induced cell death.

### 2.3. Pst_9302 Inhibits PTI in Wheat

**Figure 4:**
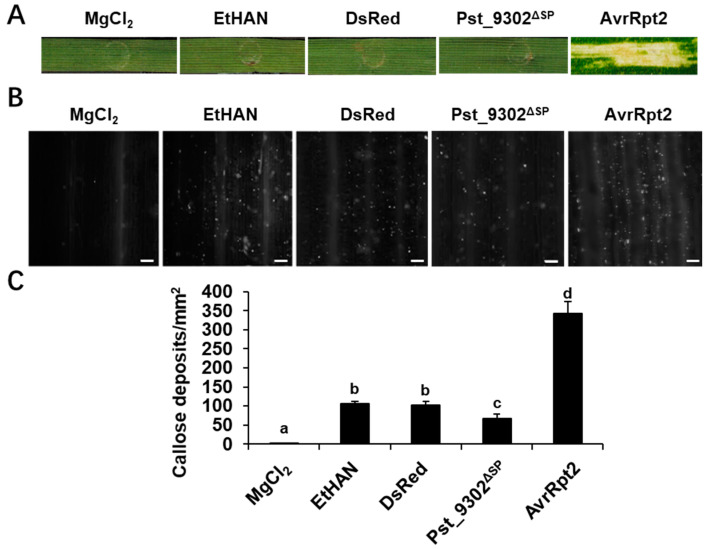
Overexpression of Pst_9302^ΔSP^ suppresses PTI-associated callose deposition in wheat leaves challenged by non-pathogenic bacteria. (**A**) Representative wheat leaves inoculated with MgCl_2_ buffer, EtHAn, or EtHAn carrying pEDV6: *DsRed*, pEDV6: *Pst_9302^ΔSP^*, and positive control pEDV6: AvrRpt2. Photos were taken at 48 h after bacterial infiltration. (**B**) Wheat leaves in (**A**) were sampled at 48 h and examined for callose deposition via epifluorescence microscopy after aniline blue staining. Bars = 10 µm. (**C**) Average numbers of callose deposits/mm^2^ in wheat leaves infiltrated with bacteria expressing the indicated genes were calculated using ImageJ 2 software. MgCl_2_, EtHAn, and EtHAn carrying *DsRed* were used as controls. Data are mean ± SD from three biological replicates. Significance was analyzed using Duncan’s new multiple range tests. Different lowercase letters indicate differences with a significance level of 0.05.

In order to elucidate the virulence function of effector Pst_9302^ΔSP^ in host wheat, *Pst_9302^ΔSP^* was cloned into the effector detector vector (DEV) pEDV6 and delivered into wheat plants via a modified Pseudomonas fluorescens EtHAn strain. EtHAn and the transformants of EtHAn carrying DsRed were used as the negative controls. Necrotic or chlorotic reactions were not observed in any of the wheat leaves infiltrated with buffer, EtHAn, EtHAn carrying DsRed, or Pst_9302^ΔSP^ (Figure 4A). Necrosis was observed in leaves infiltrated with EtHAn carrying *AvrRpt2*, an avirulent gene of *P. fluorescens*, along with abundant callose deposition (Figure 4A,B). AvrRpt2, which is known to trigger cell death in wheat, served as a positive control. The observed necrotic and callose accumulation caused by AvrRpt2 indicate success of the system. Callose deposition was observed in wheat infiltrated with EtHAn (Figure 4B), indicating that infection with non-pathogenic EtHAn triggers PTI in wheat. In wheat leaves expressing dsRed, no obvious change in callose deposition was observed compared to in the control plants infiltrated with EtHAn (Figure 4B,C). In contrast, under the same conditions, leaves inoculated with EtHAn carrying *Pst_9302^ΔSP^* exhibited less callose accumulation compared to those of the negative controls (Figure 4B,C). The results indicate that the *Pst* effector Pst_9302 could partially suppress PTI in wheat.

### 2.4. Pst_9302 Suppresses Wheat ETI

**Figure 5:**
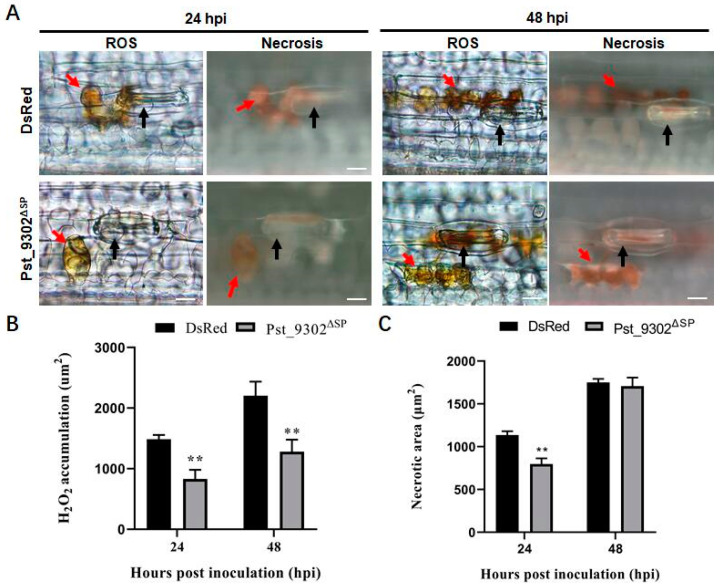
Overexpression of Pst_9302^ΔSP^ in wheat cells suppressed reactive oxygen species (ROS) accumulation and hypersensitive response triggered by the avirulent *Pst CYR23*. (**A**) The second leaves of wheat seedlings of cultivar Suwon11 were first infiltrated with EtHAn carrying Pst_9302^ΔSP^ at OD600 = 1.0 and then challenged with avirulent *Pst* race CYR23 24 h later. Inoculated leaves were sampled at 24 and 48 hpi and stained with 3,3′-diaminobenzidine for ROS detection and necrotic area at the infection sites, then observed under an Olympus BX-52 microscope. Black arrows indicate the substomatal vesicle of *Pst*, red arrows indicate the ROS and cell death area. Area of ROS (**B**) and necrotic cell death (**C**) per infection site were measured with cellSens_V4.1.1 Entry software (Olympus, Tokyo, Japan). Data are mean ± SD (*n* = 50). Significance was analyzed using an unpaired two-tailed Student’s *t*-test (**, *p* < 0.01).

Because many pathogen effectors have developed the ability to suppress ETI during evolution, we delivered Pst_9302^ΔSP^ into the wheat cultivar Suwon11 to evaluate ETI resistance to the avirulent *Pst* race CYR23. The area of reactive oxygen species (ROS) burst and necrosis area per infection site were observed and counted. DAB staining revealed that ROS accumulation per infection site in wheat leaves transiently expressing Pst_9302^ΔSP^ was reduced by 44.0% and 41.8% compared to that in wheat leaves expressing DsRed at 24 hpi and 48 hpi (Figure 5A,B). A hypersensitive response was observed under fluorescent microscopy and the fluorescence areas were calculated. Compared with the wheat leaves expressing DsRed, the necrotic cell area in wheat leaves expressing Pst_9302^ΔSP^ was decreased by 29.8% at 24 hpi (Figure 5A,C). Our results suggest that Pst_9302^ΔSP^ overexpression is able to suppress the ETI-triggered ROS accumulation of the avirulent *Pst* race in wheat.

### 2.5. Silencing of Pst_9302 Reduces Pst Pathogenicity

**Figure 6:**
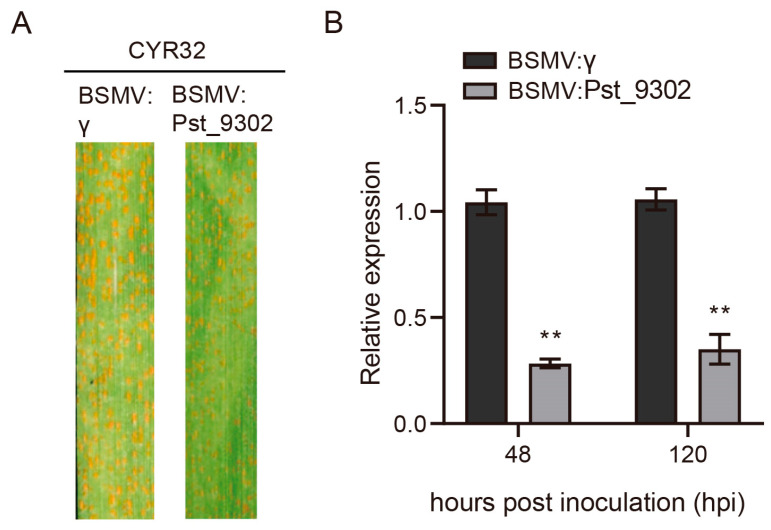
BSMV-mediated HIGS of *Pst_9302* weakened *Pst* pathogenicity. (**A**) Disease symptoms of *Pst_9302* knockdown plants inoculated with virulent *Pst* race CYR32. The fourth leaves of wheat plants inoculated with BSMV: γ and BSMV: *Pst_9302* were inoculated with *Pst* race CYR32 at 10 days post inoculation of BSMV. Disease phenotypes were observed at 14 days post inoculation of *Pst*. (**B**) Silencing efficiency of *Pst_9302* at 48 and 120 hpi analyzed via qRT-PCR. Data are means with SD from three biological replicates. **, *p* < 0.01 via an unpaired two-tailed Student’s *t*-test.

**Figure 7:**
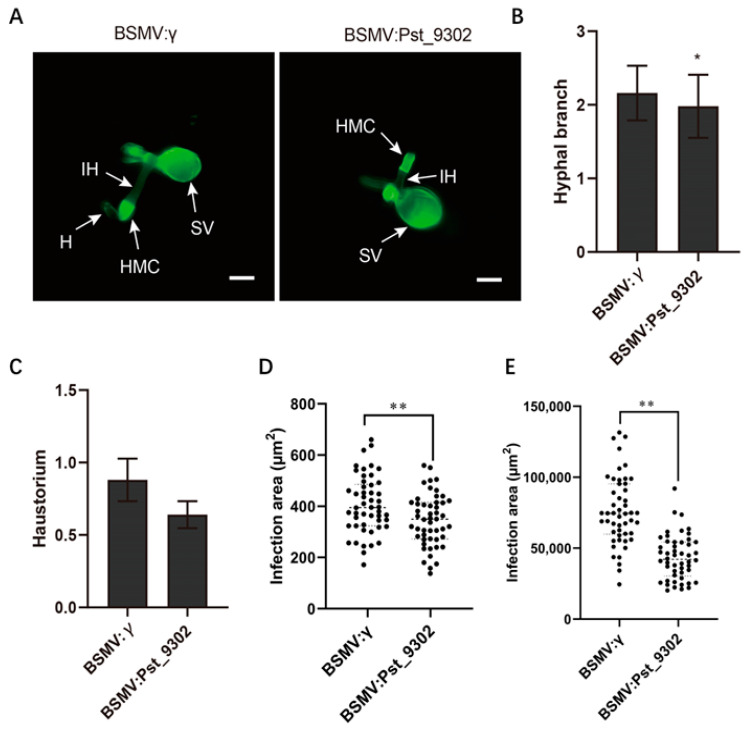
Knockdown of *Pst_9302* attenuated *Pst* growth and development. The *Pst* structures in *Pst_9302* knockdown plants and the control plants were stained with wheat germ agglutinin conjugated to Alexa 488 (WGA) at 48 hpi and 120 hpi and observed under a fluorescence microscope. (**A**) Histological observation of *Pst* growth in *Pst_9302*-silenced wheat plants at 48 hpi. SV, sub-stomatal vesicle; IH, infection hyphae; HMC, haustorial mother cell; H, haustorium. Bars = 20 µm. (**B**,**C**) The hyphal branch and haustorium numbers of *Pst* per infection site at 48 hpi were measured using CellSens Entry software (Olympus, Tokyo, Japan) (**D**,**E**) Infection area of *Pst* in *Pst_9302*-silenced wheat plants at 48 and 120 hpi measured using CellSens Entry software (Olympus, Tokyo, Japan). Data are means ± SD (*n* = 50). *, *p* < 0.05; **, *p* < 0.01 using an unpaired two-tailed Student’s *t*-test.

To further characterize the function of *Pst_9302* in *Pst* pathogenicity, we knocked down the expression of *Pst_9302* during wheat–*Pst* interaction using barley stripe mosaic virus (BSMV)-mediated host-induced gene silencing (HIGS). One specific silencing fragment of *Pst_9302* was designed to generate the recombinant virus constructs. The BSMV: γ-*TaPDS* was used as the virus indicator. At 10 days post BSMV inoculation, BSMV: γ-*TaPDS*-inoculated wheat leaves showed photobleaching, indicating the successful work of the virus system. Then, the fourth leaves of BSMV inoculated wheat plants were further challenged with *Pst* race CYR32, which was virulent on the control wheat plants. The disease phenotypes were observed at 14 dpi. BSMV: γ-inoculated wheat leaves were susceptible to *Pst* race CYR32 with abundant uredium formed, and in BSMV: *Pst_9302*-inoculated wheat leaves, decreased uredium was formed (Figure 6A). qRT-PCR analyses showed that the expression level of *Pst_9302* was reduced by 60–75% in *Pst_9302* knockdown plants compared to in the control plants (Figure 6B). Histological observation revealed that the hyphal branch per infection site was significantly reduced in *Pst_9302*-silencing wheat plants at 48 hpi compared to that in the control leaves (Figure 7A–C). At both 48 hpi and 120 hpi, the *Pst* infection areas were significantly decreased in *Pst_9302*-silencing wheat plants compared to those in the control leaves (Figure 7D,E). The data showed that knocking down the expression of *Pst_9302* weakened *Pst* pathogenicity, indicating the contribution of *Pst_9302* in stripe rust fungal virulence.

### 2.6. Pst_9302 Interacts with TaVDAC1 In Vitro and In Vivo

**Figure 8:**
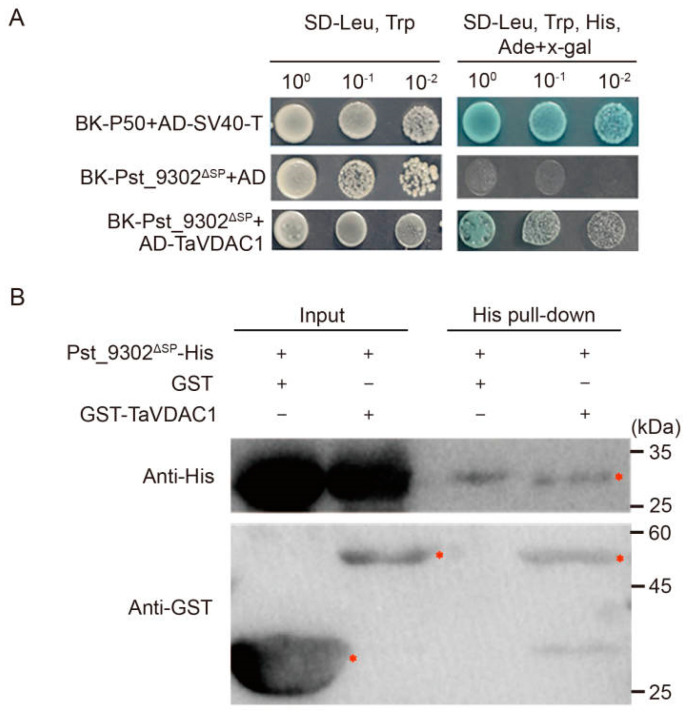
Pst_9302 interacts with TaVDAC1 in vitro and in vivo. (**A**) Interaction of Pst_9302 and TaVDAC1 by Y2H. A series of dilutions of yeast cells co-transforming BK-Pst_9302^ΔSP^ and AD-TaVDAC1 were incubated on SD-LW medium and SD-LWHA medium with X-α-gal. BK-Pst_9302^ΔSP^ and AD were used as the negative controls. SV40 large T-antigen (T) and murine p50 were used as the positive controls. (**B**) Interaction between Pst_9302-His and GST-TaVDAC1 via pull-down assay. The inputs and the immunoprecipitated protein complexes were detected via Western blot using anti-His antibody and anti-GST antibody. The red asterisks indicate the target protein.

To understand the virulence function mechanism of Pst_9302 in suppressing wheat immunity, a yeast two-hybrid (Y2H) assay was performed to identify the potential host targets of Pst_9302. With Pst_9302^ΔSP^ as the bait, 18 distinct putative wheat targets with known annotation were identified from a wheat-*Pst* cDNA library. The coding sequence of six interacting candidate genes was successfully cloned into pGADT7. The recombinant pGADT7 constructs were co-transformed with pGBKT7-Pst_9302^ΔSP^ to detect their interaction via Y2H. Yeast cells expressing Pst_9302^ΔSP^ and wheat protein encoding voltage-dependent anion channels, named TaVDAC1, grew and turned blue on the selection medium, SD/-Trp/-Leu/-His/-Ade with X-a-gal added (Figure 8A), indicating the interaction between Pst_9302 and TaVDAC1. To determine whether Pst_9302 can directly interact with TaVDAC1, a pull-down assay was conducted. GST-TaVDAC1 or GST protein was co-incubated with Pst_9302-His and precipitated using His beads. The immunoprecipitated protein complexes were detected via Western blotting using anti-His antibody and anti-GST antibody. The results showed that GST-TaVDAC1 was able to interact with His-Pst_9302, whereas GST alone could not (Figure 8B). 

## 3. Discussion

During infection, wheat stripe rust fungus secrets clusters of effectors via haustorium to host cells to regulate plant immunity and metabolism, thus promoting host plant susceptibility. Identifying the key effectors of *Pst* is of great significance in revealing the pathogenic mechanism of rust fungi. The completed *Pst* genome and haustorial transcriptome identified a large quantity of secreted proteins [24,25]. Developed functional analyses systems, including EtHAn-mediated rust fungal effector expression in wheat and HIGS, lay the foundation for analyzing the function of rust fungal effectors [35,36]. Via HIGS- and T3SS-mediated effector delivery technology, a series of *Pst* effectors that significantly affect *Pst* virulence have been identified [26,27,28,29,37,38]. In this study, one *Pst* effector, Pst_9302, which is highly induced at 18 hpi during *Pst* infection in wheat, was identified. During *Pst* infection, after landing on the wheat surface, the urediniospores germinate and penetrate through the stoma to form sub-stomatal vesicles, then the initial infection hyphae were formed at 12 hpi and began to form an initial haustorium at 18 hpi. Therefore, the high transcript level of *Pst_9302* at 18 hpi indicates the possible role of *Pst_9302* during the early establishment of the parasitic relationship of *Pst* in host plants. BlastN analyses showed that *Pst_9302* is a *Pst*-specific effector, which is conservative among *Pst* isolates, indicating that Pst_9302 may function as a common virulent factor in *Pst* but may be lacking in other rust fungi.

The ability to suppress Bax-triggered PCD is a valuable initial screen for pathogen effectors because it physiologically resembles defense-related HR [32]. Although it is possible that the use of a non-host species could result in some responses not relevant to *Pst* infection, the ability to suppress PCD triggered by Bax indicates that *Pst_9302* is able to suppress PCD in plants. *Pst_9302* is able to suppress callose deposition elicited by the non-pathogenic *Pf* strain EtHAn. Callose deposition is a hallmark of PTI. The suppression of callose deposition by *Pst_9302* indicates that it can inhibit host PTI. In fact, as the first line of defense, PTI involves multiple processes that can be attenuated by diverse pathogens to achieve further infection. Most effectors of the oomycete *Hyaloperonospora arabidopsidis* and bacterial *Psedomonas syring* pv. *tomato* DC3000 (*PtoDC3000)* can suppress PTI in plants elicited by non-pathogenic microorganisms and PAMPs in different ways [39,40,41]. For example, the *Hyaloperonospora arabidopsidis* effector HaRxL96 and its homologue PsAvh163 in *Phytophthora sojaeare* could both suppress immunity in soybean. HaRxL96 inhibits immunity in *Nicotiana benthamiana*, whereas PsAvh163 induces an HR-like cell death response in *N. benthamiana* that is dependent on RAR1 and Hsp90.1 [39]. Oomycete effector AVRblb2 interacts with cyclic nucleotide-gated channels using calcium sensors as cofactors to inhibit calcium channel activation and suppress pattern-triggered immunity [41]. Bacterial effectors are also reported to suppress plant immunity by targeting PTI signaling components. *Ralstonia solanacearum* subverts plant PTI using multiple effectors, among which RipE1 manipulates JA signaling and SA synthesis to promote infection [42]. The *Pst* effector HASP98 targets and inhibits the kinase activity of wheat TaMAPK6, the key component of the plant basal defense pathway, to suppress wheat immunity [26]. It is possible that various *Pst* effectors function by targeting different conserved components of the PTI signaling pathway.

*Pst_9302* also significantly suppress ETI responses in wheat plants challenged with the avirulent *Pst* isolate CYR23. Because biotrophs must actively suppress PCD in host plants to propagate, the suppression of ETI responses observed in this study demonstrates their virulence functions. Recent studies reported that plant PTI and ETI are not distinctive pathways but are able to potentiate each other. The inactivation of the key receptors in PTI also abolished ETI [6]. Thus, the suppression ETI by Pst_9302 may partially result from the inhibited PTI.

HIGS is a useful tool to study the function of effectors in biotrophic fungi, which enable the identification of effectors that play a majority function in pathogen pathogenicity. For example, the HIGSs of *Pst_13661*, *Pst_A23*, *Pst_4*, *Pst_5*, etc., weaken *Pst* pathogenicity and increase wheat resistance to *Pst* [12,29,43]. In our study, HIGS effectively silenced *Pst_9302*, and silenced plants produced fewer urediniospores. Infection area and hyphae branches were reduced. These results suggest that Pst_9302 functions as an important pathogenicity factor that contributes to *Pst* virulence.

The effector proteins of wheat stripe rust can regulate host immune response through various action pathways. Pst_12806, Pst_4, and Pst_5 inhibit chloroplast-mediated immunity by interfering with photosynthesis and promoting pathogenicity [28,29]. The *Pst* effector PstGSRE1 is rich in glycine and serine and regulates host gene transcription by stopping transcription factor TaLOL2 from entering into the nucleus [27]. The nuclear localized effectors Pst_A23 and PstGTA regulate host gene expression via post-transcription modification, by modulating host pre-mRNA alternative splicing or the H3K4 acetylation level of *TaSIG* to promote fungal infection [12,30]. Pst18363 and Pst27791 enhance the activity of the negative immune regulatory factor Nudix hydrolase23 (TaNUDX23) and the stability of TaRaf46, while PsSpg1 inhibits host disease resistance by increasing the kinase activity of the susceptible gene TaPsIPK1 [31,44,45]. These effectors manipulate susceptible host genes to mediate wheat susceptibility, while another type of effector protein suppresses host immunity by inhibiting the activity of immune positive regulatory factors. Hasp98 and PstGSRE4 inhibit the activity of the host immune positive regulatory kinase TaMAPK4 and the copper zinc superoxide dismutase TaCZSOD2, respectively, leading to a decrease in the accumulation of reactive oxygen species in the host [26,46]. Voltage-dependent anion channels (VDACs), a component of the outer membrane of mitochondria, were first identified in yeast and are present in all organisms [47]. VDACs regulate the transport of substances between cytoplasm and mitochondria, e.g., K^+^, Cl^−^, Ca^2+^, HPO_4_^2−^, ATP^4−^, O^2−^, and mitochondria-mediated cell death [46]. In plants, studies have shown that VDACs not only affect plant development but also positively regulate pathogen defense [48]. 

Arabidopsis AtVDAC1 regulates infection resistance to *Agrobacterium tumefaciens*. NbVDACs are involved in resistance to *Pseudomonas cichorii* [49]. Notably, VDACs also function in Bax-mediated cell death [50,51]. Given these results, we hypothesize that Pst_9302 suppresses Bax-induced cell death and plant immunity via association with plant mitochondrial VDACs. Further studies are necessary to investigate the role of TaVDAC1 in plant cell death and defense response to *Pst* infection and analyze the regulation mechanism between Pst_9302 and TaVDAC1.

## 4. Materials and Methods

### 4.1. Plant Materials, Fungal Isolates, and Bacterial Strains

Seedlings of wheat (*Triticum aestivum* L.) cultivar Suwon 11 (Su11) and *N. benthamiana* were grown in a greenhouse under 8/16 h night/day conditions at 16 °C and 22 °C, respectively. *Pst* races CYR23 and CYR32 were propagated on wheat cultivar Mingxian 169 and Suwon 11. A. *tumefaciens* strain GV3101 was cultured in LB medium at 28 °C for transient expression in *N. benthamiana*. *P. fluorescens* strain EtHAn was cultured in King’s B medium at 28 °C and used to deliver effectors to wheat. Yeast strains AH109 were cultured in YPDA medium at 30 °C for Y2H assays. *E. coli BL21(DE3)* was used for protein expression.

### 4.2. Cloning and Sequence Analysis

The sequence of *Pst_9302* was derived from the CYR32 genome. The signal peptide was identified by using SignalP 4.0 (http://www.cbs.dtu.dk/services/SignalP/, accessed on 15 May 2023). Protein domains were predicted using the Conserved Domain Search of NCBI (https://www.ncbi.nlm.nih.gov/Structure/cdd/wrpsb.cgi?, accessed on 15 May 2023). The transmembranes of proteins were analyzed using TMHMM2.0 software (http://www.cbs.dtu.dk/services/TMHMM-2.0/, accessed on 15 May 2023).

The nucleotide sequences of *Pst_9302* from 13 *Pst* isolates were downloaded from the GenBank nucleotide database, including CYR32, PST-130, PST43_v01, PST21_v01, PST-78, 38S102, Race_31, Race_K, Race_Yr9, 104_E137_A, 93TX-2, 11-281, and DK09_11. DNAMAN8.0 software was used for sequence alignment. 

### 4.3. RNA Isolation and qRT-PCR

To measure the expression levels of *Pst_9302* in the *Pst*-infected wheat leaves, the leaf tissues were sampled at 18 h, 24 h, 48 h, 72 h, 120 h, 168 h, and 216 h and stored at −80 °C for RNA extraction. Frozen urediniospores, germinated tubes, and infected leaves were ground in liquid nitrogen, and RNA was isolated with the TRIzol reagent (Invitrogen, Carlsbad, CA, USA) following the manufacturer’s instructions. RNA was reverse transcribed using a RevertAid First Stand cDNA Synthesis kit (Thermo Fisher Scientific, Waltham, MA, USA). RT-qPCR analysis was performed using ChamQ SYBR qPCR Master Mix (Vazyme, Nanjing, China) on a Bio-Rad CFX96 Real-Time PCR Detection System (Bio-Rad, Hercules, CA, USA). The elongation factor genes *PstEF1* from *Pst* was used as internal references. Primers used in qRT-PCR are listed in Table S1. Relative expression levels of target genes were calculated using the comparative 2^−∆∆CT^ method [52]. Each experiment was performed for three biological replications.

### 4.4. Plasmid Construction

For the transient expression of Pst_9302 in *N. benthamiana*, the coding sequence (CDS) of *Pst_9302* without a signal peptide and the *Bax* gene were PCR amplified and inserted into the *ClaI*/*NotI* restriction sites in vector potato virus X (PVX) pGR106 to obtain the recombinant plasmids PVX-*Pst_9302^ΔSP^* and PVX-*Bax*.

For transient expression in wheat via *Pf* strain EtHAn, sequences encoding mature proteins of Pst_9302 without the signal peptide were amplified from *Pst* race CYR32 cDNA and cloned into the pDONR221 vector (Invitrogen, Carlsbad, CA, USA). pDONR221 constructs were transferred to pEDV6 via a gateway LR recombination reaction (Invitrogen, Carlsbad, CA, USA).

For BSMV-mediated HIGSs of *Pst_9302*, a 142 bp fragment specific to *Pst_9302* was designed using siRNA-Finder21 (Si-Fi21) software analysis and ligated to *Not* I/*Pac* I-digested BSMV: γ vector to construct the RNA-based derivative plasmids BSMV-*Pst_9302*-as.

In order to screen target proteins, sequence-encoding Pst_9302 mature proteins without the signal peptide were inserted into pGBKT7 to form BK-Pst_9302. The full-length cDNA sequence of the TaVDAC1 protein was inserted into pGADT7 to form AD-TaVDAC1.

To confirm the interaction between Pst_9302 and TaVDAC1, the pull-down assay was performed. The sequence-encoding-Pst_9302^ΔSP^ protein was cloned into pET32a, and the TaVDAC1 protein was cloned into pGEX4T-1. The primers used for all constructs are listed in Table S1. 

### 4.5. A. tumefaciens Infiltration Assays for Suppression of Bax-Induced PCD

Constructs were introduced into the *A. tumefaciens* strain GV3101 via electroporation, and positive transformants were selected using kanamycin and rifampicin. Individual clones were verified via PCR. For infiltration into leaves, recombinant strains of *A. tumefaciens* were grown in LB medium for 48 h, harvested, washed three times with 10 mM MgCl_2_, resuspended in 10 mM MgCl_2_ to a final OD600 = 0.2, and incubated at room temperature for 2 h prior to infiltration. Cell suspensions were infiltrated into *N. benthamiana* leaves using a syringe without needle. To assay the suppression of Bax-induced cell death, an *A. tumefaciens* cell suspension carrying Pst_9302^ΔSP^ was initially infiltrated. *A. tumefaciens* cells carrying Bax were infiltrated into the same site 24 h later. Symptoms were scored 3–7 days post infiltration (dpi) with Bax. Each assay consisted of at least three plants and three leaves and was performed in triplicate.

### 4.6. Bacterial TTSS-Mediated Delivery of Pst_9302 in Wheat Plants

Constructs were transformed into *Pf* EtHAn via electroporation. For transient expression in wheat cells, bacteria were grown overnight, collected, washed twice with sterile 10 mM MgCl_2_, and resuspended to an OD600 = 1.0. For the analysis of PTI suppression, the suspended cells were infiltrated into the second leaf of wheat cultivar Swon11. Leaves infiltrated with EtHAn carrying DsRed were used as negative control. At 24 h post inoculation, the infiltrated wheat leaves were collected and stained with aniline blue to count callose accumulation, which was measured as an indicator of PTI.

In ETI-suppression assays, the second leaves of wheat cultivar Suwon11 were initially infiltrated with recombinant EtHAn at an OD600 = 1.0. Twenty-four hours post infiltration (hpi), infected leaves were challenged with the avirulent *Pst* race CYR23 at the initial infiltration site. Challenged leaves were sampled at 24 and 48 hpi for histochemical observation. Oxidative burst and cell death occurrence were scored to evaluate the resistance response. Leaves infiltrated with EtHAn carrying DsRed were used as negative controls and all data were compared to results for the negative controls.

### 4.7. BSMV Mediate Host-Induced Gene Silencing

BSMV constructs (BSMV: *Pst_9302* for the silencing of *Pst_9302*, BSMV: γ and BSMV: *TaPDS*) were used to inoculate the second leaves of the two-leaf stage wheat cultivar Swon11 [53]. BSMV: γ was used as the negative control, and BSMV: *TaPDS* was used as a virus-positive control. Ten days after virus inoculation, the fourth leaves were further inoculated with the fresh urediniospores of *Pst* CYR32. The inoculated leaves were collected at 48, and 120 hpi for silencing efficiency analyses and the histological observation of fungal growth. Fourteen days after *Pst* inoculation, the phenotypes of the fourth leaves were examined and photographed.

### 4.8. Histochemical Analysis

To visualize callose deposition, wheat leaves were harvested, cleared, and stained with aniline blue [54]. Briefly, the collected wheat leaves were immersed in ethyl alcohol:acetic acid (1:1 *v*/*v*) and chloral hydrate until the leaf segment was transparent, and were then stained with 0.05% aniline blue in 0.067 M K_2_HPO_4_ (pH 9.6). Stained samples were observed with an Olympus BX-52 microscope (Olympus Corporation, Tokyo, Japan) under blue light excitation (excitation wavelength 450–480 nm, emission wavelength 515 nm). The number of callose depositions was calculated for 1 mm^2^ in 50 replicates using ImageJ software. Hydrogen peroxide accumulation was monitored using (3,3′-diaminobenzidine) DAB staining as previously described [55]. The sampled *Pst*-infected leaves were incubated with 1.5 mg/mL DAB under illumination for 5 h at room temperature. H_2_O_2_ accumulation was examined and recorded with an Olympus BX-52 microscope equipped with cellSens Entry software. The autofluorescence of attacked mesophyll cells was observed under a fluorescence microscope (excitation filter 485 nm, dichromic mirror 510 nm, barrier filter 520 nm) and measured using cellSens Entry software (Olympus, Tokyo, Japan). Wheat germ agglutinin (WGA) conjugated to Alexa 488 (Invitrogen) was used to stain the infection structure of *Pst* as previously described [56]. The hyphal branch was measured as the number of observed infection hyphae formed from one substomatal vesicle (SV). The haustoria was measured as the number of observed haustoria formed at the tip of all infection hyphae from one SV. The infection area was calculated as the area of infection hyphae using CellSens Entry software (Olympus, Tokyo, Japan). Using cellSens Entry software (Olympus, Tokyo, Japan), the number of haustoria, hyphae branches, and hyphal infection areas were measured from 50 infection sites in which substomatal vesicles had formed underneath the stoma.

### 4.9. Yeast Two-Hybrid Assay

The recombinant Pst_9302^ΔSP^-pGBKT7 vector was used as a bait to screen a Y2H cDNA library produced from *Pst*-infected wheat leaves. Yeast strain AH109 co-transformed with BK-*Pst_9302^ΔSP^* and the library plasmids were cultured on SD/-Trp-Leu and screened on SD/-Trp-Leu-His-Ade and X-a-gal-supplemented medium. Positive colonies on SD/-Trp-Leu-His-Ade plates were used as templates for PCR, and the products were sequenced. The coding sequences of the candidate interacting proteins were cloned into pGADT7 and co-transformed with BK-*Pst_9302^ΔSP^* into yeast strain AH109 to examine their interactions through growth on the SD/-Trp-Leu-His-Ade and X-a-gal-supplemented medium. 

### 4.10. Protein Expression in E. coli and Purification

The recombinant plasmids PET32a-*Pst_9302^ΔS^*^P^ and PGEX4T-1-*TaVDAC1* were transformed into *E. coli BL21 (DE3)*. A single colony that had been freshly plated was inoculated into 40 mL of liquid LB media before undergoing shaking at 200 rpm at a temperature of 37 °C Then, the 40 mL culture was inoculated into 1 L of LB and cultured at 37 °C to OD_600_ = 0.6. Then, 0.5 mM IPTG was added to the culture before undergoing additional shaking at 160 rpm at a temperature of 18 °C for 20 h. *E. coli* cells were harvested and resuspended in 50 mM Tris (pH 7.5), 150 mM NaCl, 20 mM *β*-mercaptoethanol (β-ME), and 1 mM PMSF and lysed via sonication. The lysates were centrifuged for 20 min at 20,000× *g*. The His-Pst_9302^ΔSP^ and GST-TaVDAC1 were purified via affinity chromatography using a HisTrap (Ni-NTA) column (GE Healthcare, Chicago, IL, USA) and GST-S Glutathione Sepharose 4B chromatography (GE Healthcare).

### 4.11. Pull-Down Assay

The interactions between His-Pst_9302^ΔSP^ and GST-TaVDAC1 were assessed via pull-down assays using a HisTrap (Ni-NTA) column (GE) according to the manufacturer’s instructions. The input proteins and proteins that had been immunoprecipitated via Ni-NTA beads (His pull-down) were analyzed through immunoblotting with anti-GST-tag mouse polyclonal antibodies (Beyotime, Shanghai, China) and anti-His-tag mouse monoclonal antibodies (Beyotime, Shanghai, China).

## Data Availability

The data presented in this study are available on request from the corresponding author.

## Supplementary Materials

The following supporting information can be downloaded at: https://www.mdpi.com/article/10.3390/plants13010094/s1, Figure S1: Prediction of the signal peptide of Pst_9302; Figure S2: Interaction between Pst_9302 and its putative targets analyzed by Y2H; Table S1: Primers used in this study; Table S2: Putative targets of Pst_9302 identified by Y2H assay.
