# Supplementary material for: Stripe Rust Effector Pst_9302 Inhibits Wheat Immunity to Promote Susceptibility
Source: Plants (Basel). 2023 Dec 27;13(1):94. doi: 10.3390/plants13010094 (PMC10780974; doi:10.3390/plants13010094)
Supplement: Supplementary file 1 [file plants-13-00094-s001.zip › plants-2782555-supplementary.pdf]

Supporting Information

Article title: Stripe Rust Effector Pst_9302 Inhibits Wheat Immunity to Promote Susceptibility

Author: Haibin Zhao, Jiangyu Huang, Xiaoyan Zhao, Ligang Yu, Xiaodong Wang, Congcong Zhao, Hojjatollah Rabbani nasab, Chunlei Tang * and Xiaojie Wang *

Figure S1 Prediction of the signal peptide of Pst_9302

Figure S2 Interaction between Pst_9302 and its putative targets identified by Y2H

Table S1 Primers used in this study

Table S2 Putative targets of Pst_9302 identified by Y2H assay

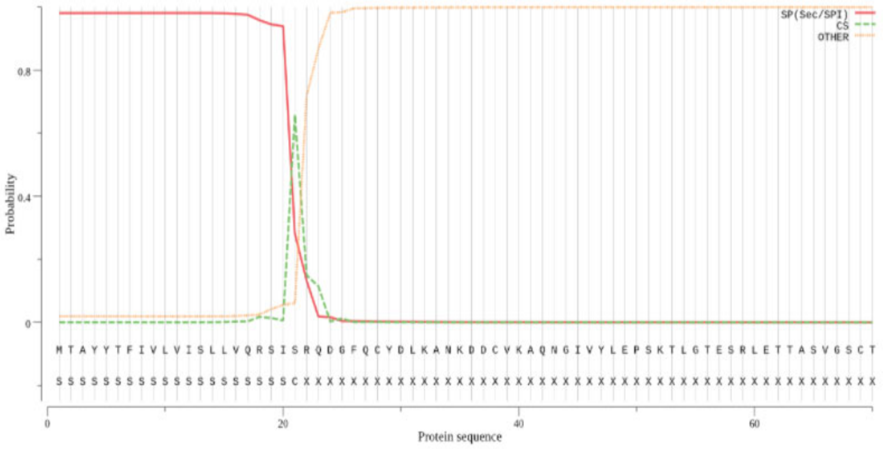

Figure S1 Prediction of the signal peptide of Pst_9302

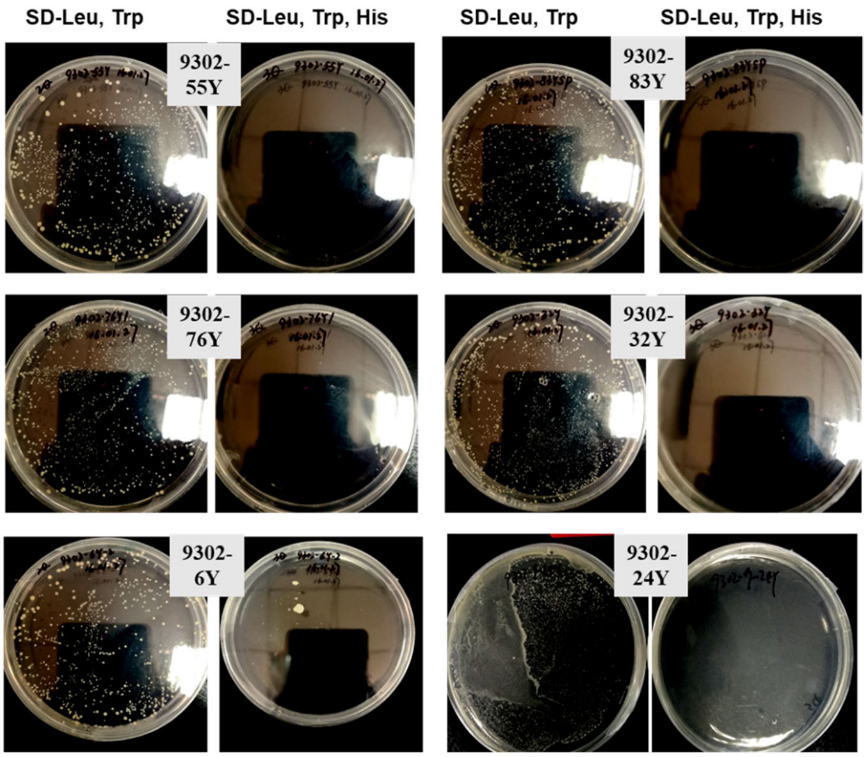

Figure S2 Interaction between Pst_9302 and its putative targets identified by Y2H. 2Q represents the SD/−Trp−Leu medium and 3Q represents SD/−Trp−Leu−His medium. The 6 genes including Oryzain alpha chain (named 9302-55Y), Heat shock cognate 70 kDa protein 1 (9302-32Y), Bark storage protein A (9302-83Y), putative protein phosphatase 2C 41 (9302-76Y), membrane magnesium transporter (9-24Y), and Voltage-dependent anion-selective channel protein (9-6Y).

Table S1 Primers used in this study.

Primer name	Sequence (5'-3')	Purpose
qRT-PsEF-F	TTCGCCGTCCGTGATATGAGACAA	
qRT-PsEF-R	ATGCGTATCATsGGTGGTGGAGTGA	qRT-PCR
qRT-Pst_9302-F	CGACTTGAAACAACTGCGT	
qRT-Pst_9302-R	GCCTTTGCGGCGATA	
PVX-Pst_9302$^{\Delta SP}$-F	gtacccggg AGGCAAGATGGATTCC	
PVX-Pst_9302$^{\Delta SP}$-R	ataagaatgcggccgcGTAATTCGAAGGCATA	
PVX-Bax-F	gtacccgggATGGACGGGTCCGGG	
PVX-Bax-R	ataagaatgcggccgcGCCCATCTTCTTCCAG	Suppress PCD
pGBKT7-Pst_9302$^{\Delta SP}$-F	aggaggacctgcatatg AGGCAAGATGGATTCC	
pGBKT7-Pst_9302$^{\Delta SP}$-R	cggatccccgggaattc GTAATTCGAAGGCATA	
pGADT7-TaVDAC1-F	ccagattacgctcatatgATGGGCGGCCCAGGCCTCT	
pGADT7-TaVDAC1-R	cccacccgggtggaattcAGGCTTGAGAGCAATA	
pET32a-Pst_9302$^{\Delta SP}$-F	GGATCCAGGCAAGATGGATTCC	Pull-down assay
pET32a-Pst_9302$^{\Delta SP}$-R	CTCGAGGTAATTCGAAGGCATA	
pGEX4T-1-TaVDAC1-F	GGATCCATGGGCGGCCCAGGCCTCT	
pGEX4T-1-TaVDAC1-R	CTCGAGTTAAGGCTTGAGAGCAATA	
Higs-Pst_9302-F	tagctagctgattaattaaGCTTATTACACATTT	
Higs-Pst_9302-R	ttgctagctgagcggccgcACTCTCCGTACCAAG	TTSS
pEDV6- Pst_9302$^{\Delta SP}$-F	ggggacaagtttgtacaaaaaagcaggcttc AGGCAAGATGGATTCC	
pEDV6- Pst_9302$^{\Delta SP}$-R	ggggaccactttgtacaagaaagctgggtc GTAATTCGAAGGCATA	

Table S2 Putative targets of Pst_9302 identified by Y2H assay.

	Annotation	Genebank	Species	Counts
1	Chloroplast-localized PtrTox A-binding protein 1	EMT15486.1	*Triticum aestivum*	3
2	**Oryzain alpha chain**	EMS56635.1	*Triticum urartu*	2
3	endoglucanase 11	EMS60957.1	*Aegilops tauschii*	1
4	**Heat shock cognate 70 kDa protein 1**	EMS51616.1	*Triticum urartu*	4
5	**Voltage-dependent anion-selective channel protein**	P46274.1	*Triticum aestivum*	1
6	Proteasome subunit alpha type-6	BAJ85020.1	*Hordeum vulgare subsp. Vulgare*	6
7	**WRKY transcription factor 3**	ABR87001.1|	*Hordeum vulgare subsp. Vulgare*	2
8	Elicitor-responsive protein 1	EMS48714.1	*Triticum urartu*	2
9	Potassium transporter 7	EMT06515.1	*Aegilops tauschii*	1
10	**membrane magnesium transporter**	XP_003579044.1	*Brachypodium distachyon*	1
11	ribosomal protein S20	AFM95250.1	*Triticum aestivum*	1
12	Putative LRR receptor-like serine/threonine-protein kinase	EMT31498.1	*Aegilops tauschii*	1
13	cysteine protease(CP)	EMS59857.1	*Triticum aestivum*	1
14	**putative protein phosphatase 2C 41**	EMS49328.2	*Triticum urartu*	3
15	Photosystem II 10kDa polypeptide, chloroplastic	EMS68597.1	*Triticum urartu*	1
16	**Bark storage protein A**	EMT26191.	*Aegilops tauschii*	1
17	D-glycerate 3-kinase,chloroplastic	XP_003569572.1	*Brachypodium distachyon*	1
18	Myb family transcription factor APL	EMT23891.1	*Aegilops tauschii*	1
19	Hypothetical protein F775_09657	EMT09722.1	*Aegilops tauschii*	2
20	hypothetical protein TRIUR3_11299	EMS60957.1	*Triticum urartu*	1
21	hypothetical protein TRIUR3_30517	EMS46308.1	*Triticum urartu*	1
22	hypothetical protein F775_07261	EMT04007.1	*Aegilops tauschii*	1

23	hypothetical protein TRIUR3_31004	EMS52954.1	*Triticum urartu*	1
24	hypothetical protein PSTG_16012	KNE90550.1	*Puccinia striiformis*	2
25	hypothetical protein PSTG_14684	KNE91886.1	*Puccinia striiformis*	2
